# Influence of Timing and Predicted Risk on Mortality in Impella-Treated Infarct-Related Cardiogenic Shock Patients

**DOI:** 10.3389/fcvm.2020.00074

**Published:** 2020-05-14

**Authors:** Andreas Schäfer, Nikos Werner, Daniel Burkhoff, Jan-Thorben Sieweke, Andreas Zietzer, Maryna Masyuk, Nanna Louise Junker Udesen, Ralf Westenfeld, Jacob Eifer Møller

**Affiliations:** ^1^Cardiac Arrest Center & Advanced Heart Failure Unit, Department of Cardiology and Angiology, Hannover Medical School, Hanover, Germany; ^2^Department of Cardiology, University Heart Center, Bonn, Germany; ^3^Department of Cardiology, Heart Center Trier, Krankenhaus der Barmherzigen Brüder, Trier, Germany; ^4^Cardiovascular Research Foundation, New York, NY, United States; ^5^Department of Cardiology, Pulmonology and Vascular Medicine, Heinrich Heine University, Düsseldorf, Germany; ^6^Department of Cardiology, University Hospital Odense, Odense, Denmark

**Keywords:** cardiogenic shock, acute myocardial infarction, ventricular assist device, acute heart failure, microaxial pumps

## Abstract

**Background:** In-hospital mortality in acute myocardial infarction-related cardiogenic shock (AMI-CS) remains high. The only adequately powered randomized trial showed no benefit of routine use of the intra-aortic balloon pump in AMI-CS. We compared individually predicted mortality using CardShock- and IABP-Shock II-scores in AMI-CS patients treated with an Impella microaxial pump, who met the IABP-Shock II-trials inclusion/exclusion criteria, to observed mortality on circulatory support in order to determine whether standardized use of an Impella microaxial flow-pump in AMI-CS is associated with lower than predicted mortality rates and whether timing of implantation or selecting patients based on predicted risk is meaningful.

**Methods and Results:** We analyzed data from 166 consecutive Impella-treated AMI-CS patients meeting the inclusion/exclusion criteria of the IABP-Shock II-trial (age 64 ± 11 years). Thirty-nine percentage of 64 patients had been resuscitated before Impella implantation. Overall 30-day mortality was 42%. Mortality was higher in resuscitated patients (50 vs. 36%, *p* = 0.0452) and when Impella was implanted post-PCI (Impella-pre-PCI: 28%, Impella-post-PCI: 51%, *p* = 0.0039). While in both score systems there was no significant difference between predicted and observed overall 30-day mortality, predicted mortality was significantly higher than observed mortality on Impella support only for individuals with highest predicted risk based on CardShock score (predicted 77 vs. observed 51%, *p* = 0.025).

**Conclusions:** Our retrospective analysis suggests that the use of the Impella microaxial pump may be effective in selected cases of high risk patients with AMI-CS.

**Condensed abstract:** Mortality is high in acute myocardial infarction-related cardiogenic shock despite rapid revascularization. Haemodynamic support with an intraortic balloon pump does not reduce mortality. In this retrospective registry including 166 consecutive IABP-Shock II-eligible cardiogenic shock patients in four dedicated shock centers, observed mortality on circulatory support with an Impella was significantly lower than predicted in patients with highest mortality risk. Implantation prior to PCI in acute myocardial infarction-related cardiogenic shock seemed to be associated with lower mortality than implantation post PCI.

## Introduction

Cardiogenic shock (CS) may be caused by several conditions, though acute myocardial infarction (AMI) is one of the major contributors (1). The “Should We Emergently Revascularize Occluded Coronaries for Cardiogenic Shock” (SHOCK) trial demonstrated that urgent invasive assessment and revascularization improves long term survival (2) and is therefore recommended by current society guidelines (3). However, despite almost two decades of additional research and therapeutic advances, CS is still associated with high mortality regardless of etiology, ranging between 40 and 60% (2–7).

There has been renewed interest over the past decade in the use of mechanical circulatory support (MCS) to treat CS with the goal of improving outcomes. Although the intra-aortic balloon pump (IABP) was the most frequently used MCS device, this mode of support failed to improve survival compared to standard medical therapy (4, 8) and its use is no longer recommended for routine use (Class IIIA in the ESC Guidelines) (3). Several, more powerful MCS devices are now available, including Impella, TandemHeart and extracorporeal membrane oxygenation (ECMO) (9). However, with the lack of prospective randomized data, current guidelines for use of MCS in AMI-CS are based on expert opinion and generally do not favor one system over another (3, 10).

Previous randomized clinical trials using Impella microaxial pumps in AMI-CS were underpowered to detect clinically relevant differences in outcome, observed mortality was impacted by high proportions of patients with out-of-hospital cardiac arrest (OHCA), and lacked of standardization of timing of Impella placement (11, 12). Several non-randomized registry studies have better outcomes when Impella support is initiated prior to PCI and applied in patients who have not experienced a cardiac arrest (13–18). Even though such studies do not provide definitive proof, studies such as these have formed the basis for recently introduced treatment algorithms [such as the National Cardiogenic Shock Initiative registry (19)] designed to optimize outcomes in patients with CS. Whether such approaches yield better outcomes is being tested in the currently ongoing, fully powered, prospective DanGer-Shock trial (20). Since DanGer-Shock is expected to require an additional 3 years to complete, dissemination of pertinent information that can inform clinical decision-making is of primary importance.

When retrospective data from Impella-treated patients meeting the entry criteria for the IABP-Shock II study was recently used for a matched pair analysis by many European contributing centers, no benefit of Impella compared to control/IABP was observed (21). Nevertheless, there was unpredictable deselection of Impella-treated patients from the analysis mainly due to unavailable data on LV function in IABP-Shock II patients.

In a recent small, single center analysis of patients treated with Impella CP who meet the entry criteria for the IABP-Shock II study from the HAnnover Cardiac Unloading REgistry (HACURE) (17), mortality rate was substantially lower than reported for patients treated either conservatively or with IABP (4) when they were treated by a dedicated algorithm for patients with either OHCA and/or CS (22). In order to achieve a more generalizable result and exclude single-center bias, we now collected registry data from three German and one Danish high-volume, highly experienced shock centers running Impella programs and identified an overall cohort of 166 IABP-Shock II-eligible patients treated with Impella CP or Impella 2.5. We determined mortality predicted by two recently introduced and validated scoring systems [the IABP-Shock II score (23) and the CardShock score (5)] and compared predicted to observed mortality. We further examined the impact of support when patients were stratified according to low, intermediate and high risk, according to timing of Impella use (pre- or post-Impella) and according to the occurrence of cardiac arrest prior to support.

## Methods

### Study Design

This was a retrospective, observational analysis that included data from all patients undergoing implantation of an Impella microaxial flow-pump in all four centers who matched the inclusion/exclusion criteria of the IABP-Shock II trial [as described in detail elsewhere (4)] from January 2013 to June 2016. De-identified data were entered into a combined database. All data were collected in accordance with the Declaration of Helsinki and approved by the local ethics committee of each center. Observed rates of mortality were compared to those estimated from the CardShock and IABP-Shock II scores. During the period analyzed in the current manuscript 483 patients were treated with Impella devices in the participating centers, of which 315 (65%) had AMI-CS. Of those, 166 (53% of AMI-CS, 34% of all Impella-treated patients) met the inclusion/exclusion criteria of the IABP-Shock II trial and were selected for the current analysis.

In general, all participating centers use algorithms for AMI-CS aiming for rapid detection and treatment of cardiogenic shock. Patients with AMI are taken to the cath labs when in shock and rapid revascularisation and initiation of mechanical circulatory support is used in patients requiring higher amounts of vasopressors and inotropes in conjunction with increased levels of serum lactate as sign of systemic hypoperfusion when LV-EF is impaired. Impella implantation is initiated during the initial cath lab procedure and patients are not taken to ICU for prolonged waiting periods.

### Patient Population

All 166 AMI-CS patients included in the analysis met all the inclusion and none of the exclusion criteria of the IABP-Shock II-study (4) and had been supported with either an Impella CP (*n* = 127) or an Impella 2.5 (*n* = 39) at four different shock centers in Germany (University hospitals in Hannover, Bonn and Düsseldorf) and Denmark (Odense). By applying the exact definition from IABP-Shock II, patients with OHCA were included if they had return of spontaneous circulation within 30 min. Data from these patients had already been included in local databases from 2013 to early 2017. A 29 patients from Hannover and 67 patients from Odense have been included in recent single center analyses of Impella CP, which covered larger patient populations than the specific ones reported in the present manuscript (17, 24). Patient level data of the subjects analyzed in the current manuscript had been submitted for potential matched-pair analysis with the IABP-Shock II data (21), but a large proportion of patients had been deselected before the analysis due to missing matching partners owed predominantly to unavailable LV-EF data in a larger proportion of patients from IABP-Shock II.

CardShock (5) and IABP-Shock II (23) scores were calculated for each individual patient and observed mortality for the cohort was compared to that predicted by the two scores. Of note, IABP-Shock II score provides an estimate of mortality at 30-days while the CardShock score provides an estimate of in-hospital mortality with a median of 12 days.

Since we previously demonstrated higher mortality in CS patients who had been successfully resuscitated (17), the analysis was stratified based on the presence or absence of cardiac arrest prior to Impella implantation. Additionally, since prior studies also indicated lower mortality if hemodynamic support was initiated pre-PCI (13, 14), patients were also stratified according to the timing of initiating Impella support.

### Data Collection and Definitions

Basic demographic data, the cause of CS, laboratory data and documented complications during in-hospital stay were collected. CS was defined as hypotension (systolic blood pressure <90 mmHg or need for inotropes or vasopressors to maintain systolic blood pressure >90 mmHg) and evidence of end organ hypoperfusion as indicated by altered mental status, clammy skin, or elevated lactate (>2 mmol/l) after adequate fluid resuscitation. Individual IABP-Shock II scores were based on: Age >73, history of prior stroke, serum glucose >191 mg/dl, serum creatinine >1.5 mg/dl, blood lactate >5 mmol/L, and TIMI flow <3 after PCI as detailed previously (23). Individual CardShock scores were based on etiology of shock, age, previous myocardial infarction, prior coronary artery bypass, mental status changes, LVEF, and blood lactate levels as detailed previously (5). The individual variables were fully available for all patients. Bleeding was defined by GUSTO criteria (25) and hemolysis during Impella support was defined as LDH ≥ 1000 IU/l and haptoglobin <0.3 g/l in 2 consecutive blood samples within 24 h.

### Clinical Follow-Up

Patient follow-up was for the period of hospitalization, and vital status was determined from medical records. The follow-up of those patients who were discharged from hospital before 30 days was obtained by documents of primary care physicians or rehabilitation hospitals. In case of discharge from hospital or rehabilitation within 30 days, further follow-up was performed by phone. Vital status was confirmed on all patients so that no patient was lost to follow up (17).

### Statistical Analysis

Numbers are given as *n* (%), mean ± standard deviation (SD) for normally distributed variables, or median and interquartile range (IQR) for non-normally distributed variables. Statistical analysis was performed with ANOVA and corrected for multiple comparisons with a Bonferroni correction; Kruskal-Wallis-Test was used for non-parametric tests (17). Chi-square tests were used to compare patient characteristics. Cumulative mortality was estimated by Kaplan-Meier analysis and compared between groups by the log-rank test.

Univariate Cox proportional hazard regression analyses performed with variables potentially associated with mortality rates were performed to identify factors associated with risk of death. Factors considered included: CardShock score, IABP Shock II score, resuscitation before Impella implantation and Impella implantation prior to PCI. Then, stepwise multivariate Cox regression analyses that included variables that were significantly linked to mortality in the respective univariate analyses (*p* < 0.05) were performed. Analysis for correlation and multicollinearities were performed before multivariate regressions analysis. In respect of possible multicollinearities between Card Shock Score and Card Shock Categories, Card Shock Categories were not considered for regressions analysis. Results from regression analyses are expressed as hazard ratios (HR) including 95% confidence interval (CI). Data were analyzed using GraphPad Prism 6.0 (GraphPad Software, Inc., La Jolla, CA) and SPSS Statistics 24 (IBM SPSS Statistics 24). A *p* < 0.05 was considered statistically significant.

## Results

### Patient Characteristics

The overall patient population consisted of 166 AMI-CS patients that met the IABP-Shock II entry criteria who had been treated with an Impella device. Patient characteristics are summarized in Table 1 and, for reference, values of available baseline characteristics are provided for patients enrolled in the original IABP-Shock II trial (4) and the CardShock cohort used for validation of the IABP Shock II score (23). Mean age in our cohort was 65 ± 12 years and 83% were male. Cardiac arrest occurred in 64 patients (39%) prior to Impella implantation. Impella was implanted pre-PCI in 68 patients (41%). The type of AMI was STEMI in 69% and NSTEMI in 31%. Median IABP-Shock II score was 3 (IQR 2, 4) and median CardShock score was 5 (IQR 4, 6).

**Table 1:** Baseline and procedural characteristics of the present prospective cohort.

	**Prospective cohort *n* = 166**	**IABP-Shock II (score cohort) *n* = 480**	**Card-Shock ACS *n* = 137**
Age, mean (SD), years	65 ± 12	70 [58–77]	68 [61–76]
Gender- male, *n* (%)	137 (82.5)	331 (69)	106 (77)
Height, mean (SD), cm	173 ± 12	174 [167–180]	171 [165–176]
Weight, mean (SD), [kg]	81 ± 17	80 [73–90]	78 [70–85]
BMI, mean (SD), kg/m^2^	27 ± 4	27 [25-30]	26.8 [24-27]
IABP Shock II Score, median (IQR)	3 [2-4]	3	2
0–2, low-risk, *n* (%)	72 (43)	235 (49)	81 (59)
3–4, intermediate-risk, *n* (%)	79 (48)	181 (38)	35 (26)
5–9, high-risk, *n* (%)	15 (9)	64 (13)	21 (15)
CardShock Score, median (IQR)	5 [4-6]		
0–3, low-risk, *n* (%)	19 (11)		
4–5, intermediate-risk, *n* (%)	72 (44)		
6–9, high-risk, *n* (%)	75 (45)		
Admission lactate, mean (SD), mmol/L	6.8 ± 0.5	3.7 [2.1–7.3]	4.3 [1.8–5.2]
**Pre-existing conditions**			
Hypertension, *n* (%)	100 (60)	341/477 (35)	81 (42)
Diabetes mellitus, *n* (%)	45 (27)	162/478 (34)	39 (29)
Hyperlipidaemia, *n* (%)	63 (38)	201/476 (42)	64 (47)
Smoking, *n* (%)	81 (49)	165/475 (35)	58 (42)
CKD, *n* (%)	25 (15)	–	–
LV-EF, mean (SD), %	21± 10	35	–
Cardiac arrest prior to Impella, *n* (%)	65 (39)	209/480 (44)	39 (29)
ROSC, mean (SD), min	20 ± 16	–	–
**Infarct location**, ***n*** **(%)**			
Left main	43 (26)	9%	
LAD	78 (47)	43%	
LCX	17 (10)	17%	
RCA	25 (15)	26%	
Bypass graft	3 (2)	3%	
**Revascularisation**, ***n*** **(%)**			
None	8 (5)	0%	0%
PCI	156 (94)	100%	100%
CABG	2 (1)	0%	0%
**TIMI flow at the end of procedure**			
TIMI 0/I	13 (8)		
TIMI II	15 (9)		
TIMI III	138 (83)	295 (82)	98 (72)
Bridge to durable LVAD	3 (2)		

*BMI, body mass index; CABG, coronary artery bypass graft; CKD, chronic kidney disease; LAD, left anterior descending coronary artery; LCX, left circumflex coronary artery; LVAD, left ventricular assist device; LV-EF, left-ventricular ejection fraction; PCI, percutaneous coronary intervention; RCA, right coronary artery; ROSC, Return of spontaneous circulation; SAPS II, Simplified Acute Physiology Score-II; TIMI, thrombolysis in myocardial infarction*.

Impella 2.5 was used in 39 patients (23%) during the early portion of the enrolment period, whereas Impella CP was used in 127 patients (77%) during the latter portion of the enrolment period. Impella CP patients trended to be younger and have a higher rate of pre-Impella cardiac arrest; otherwise, these patients had similar baseline characteristics. Mean support time on Impella was 3 ± 3 days. Clinically meaningful differences between the current cohort and the IABP-Shock II cohort included higher serum lactates and lower ejection fractions.

Adverse events are summarized in Table 2. The most frequent adverse events were the need for renal replacement therapy, bleeding, sepsis, and haemolysis.

**Table 2:** 30-day adverse events.

	**All *n* = 166**
Reinfarction	8 (5%)
Definite stent thrombosis	3 (2%)
Ischemic stroke	1 (1%)
Haemorrhagic stroke	3 (2%)
Peripheral ischemia of the leg requiring Surgery or intervention	15 (9%)
Haemolysis	31 (19%)
**Bleeding (based on GUSTO definitions** **(****25****))**	
Life-threatening/severe	9 (5%)
Moderate	27 (16%)
Mild	33 (20%)
Sepsis	50 (30%)
Renal replacement therapy	84 (51%)

### Observed vs. Predicted Mortality

**Figure 1:**
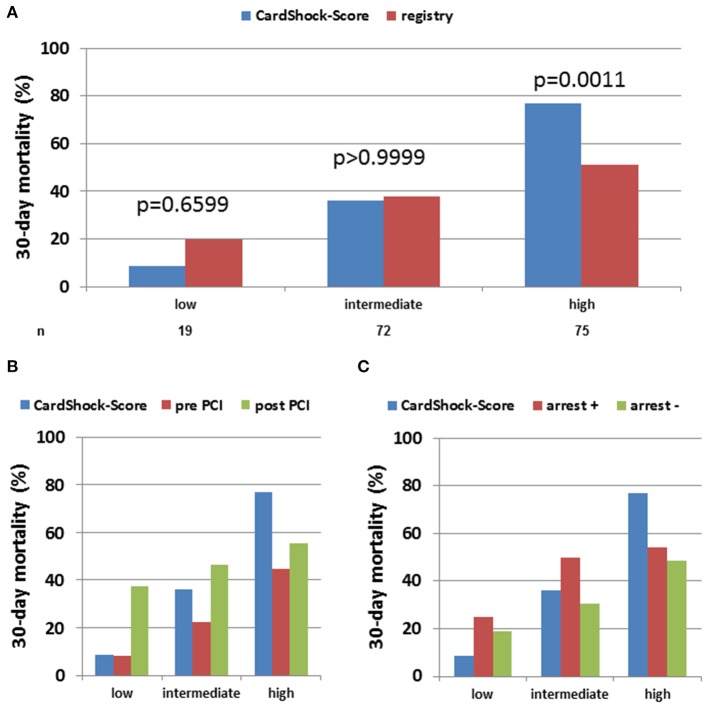
Observed vs. CardShock score-predicted 30 days in-hospital mortality in acute myocardial infarction cardiogenic shock (AMI-CS) on Impella: Observed 30-day mortality on Impella-treated AMI-CS patients was compared to predicted mortality using CardShock-score and categorized by low, intermediate or high risk based on score definition. The assessment was performed in the overall cohort **(A)** as well as after stratification for Impella implantation pre vs. post PCI **(B)** or cardiac arrest prior to Impella **(C)**.

**Figure 2:**
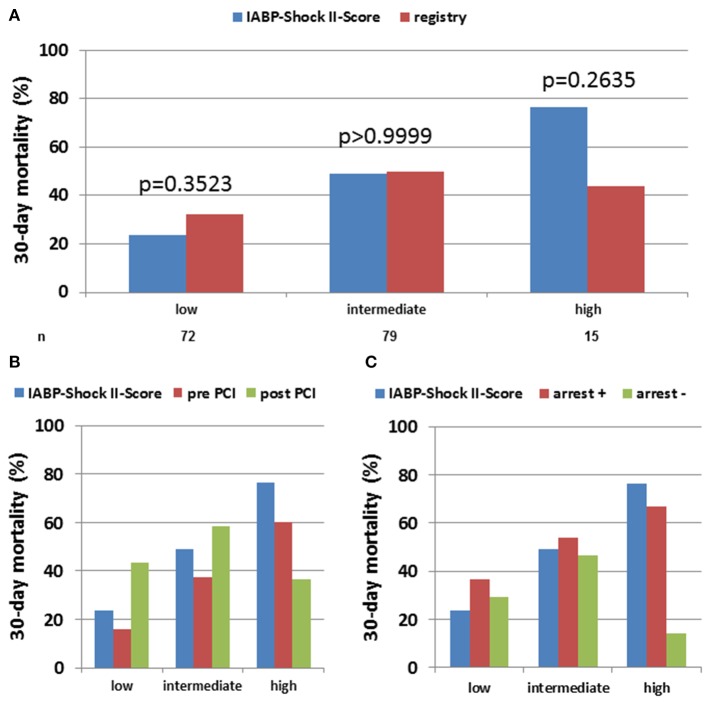
Observed vs. IABP Shock-II score-predicted 30 days in-hospital mortality in acute myocardial infarction cardiogenic shock (AMI-CS) on Impella: Observed 30-day mortality on Impella-treated AMI-CS patients was compared to predicted mortality using IABP Shock II-score and categorized by low, intermediate or high risk based on score definition. The assessment was performed in the overall cohort **(A)** as well as after stratification for Impella implantation pre vs. post PCI **(B)** or cardiac arrest prior to Impella **(C)**.

The median CardShock score was 5 which corresponds to a predicted in-hospital mortality of 40%, the median IABP-Shock II score was 3 which corresponds to a 30-day mortality of 49%, while observed 30-day mortality in our cohort was 43% (*p* = 0.12). However, since both scores use a maximum of 9 points each, but categorize only to three levels, we performed further analyses in each risk category: to gain insights into correlates of mortality, patients were sub-grouped according to low risk (IABP-Shock II score 0–2, CardShock score 0–3), intermediate risk (IABP-Shock II score 3-4, CardShock score 4-5) or high risk (IABP-Shock II score 5–9, CardShock score 6–9) scores (Figures 1A, 2A). For both scoring systems, overall observed 30-day mortality for patients with low and intermediate risk scores were similar to the respective predicted mortalities. For patients at high risk, observed mortality during Impella support was significantly lower than predicted with CardShock [predicted 77% vs. observed 51%, *p* = 0.0011, OR 0.30, 95% CI (0.15–0.61)], while observed mortality was only numerically lower using IABP-Shock II-score (predicted 77% vs. observed 44%, *p* = 0.2635, OR 0.32, 95% CI (0.07–1.47) due to the much lower number of patients categorized as high risk by IABP-Shock II- compared to CardShock-score).

Comparing characteristics of 30-day survivors and non-survivors showed that survivors were younger, had lower admission lactate levels, lower shock score values, and trended to be less often resuscitated. Importantly, there was no difference regarding type of Impella or renal function between survivors and non-survivors (Table 3).

**Table 3:** Characteristics and outcomes of 30-day survivors vs. non-survivors.

**Parameter**		**Overall cohort (*n* = 166)**	**30 day mortality (*****n*** **=** **166)**		**P survivors vs. non-survivors**
			**Survivors (*n* = 97)**	**Non-survivors (*n* =69)**	
CardShock Score		5 [4-6]	5 [4-6]	6 [5-6]	**0.001**
CardShock category					**0.025**
	Low (0–3)	19 (11%)	15 (16%)	4 (6%)	
	Intermediate (4–5)	72 (43%)	45 (46%)	27 (39%)	
	High (6–9)	75 (46%)	37 (38%)	38 (55%)	
IABP Shock II Score		3 [2-4]	3 [1-4]	3 [2-4]	**0.029**
IABP Shock II category					0.069
	Low (0–2)	72 (43%)	49 (51%)	23 (33%)	
	Intermediate (3/4)	79 (48%)	40 (41%)	39 (57%)	
	High (5–9)	15 (9%)	8 (8%)	7 (10%)	
Cardiac arrest prior to Impella		64 (39%)	32 (33%)	32 (46%)	0.057
Impella, type of device					0.693
	2.5	39 (23%)	24 (25%)	15 (22%)	
	CP	127 (77%)	73 (75%)	54 (78%)	
Impella Implantation					**0.008**
	Pre PCI	68 (41%)	49 (51%)	19 (28%)	
	Post PCI	98 (59%)	48 (49%)	50 (72%)	
Age		63.9 ± 11.3	63.5 ± 12.4	68.5 ± 10.4	**0.008**
History of stroke		14 (8%)	8 (8%)	6 (9%)	0.86
Creatinine [mg/dl]		1.36 [1.10–1.70]	1.33 [1.11–1.64]	1.47 [0.98–1.80]	0.80
eGFR [mL/min/1.73 m^2^]		32 [20-50]	32 [19-51]	31 [20-49]	0.78
Admission arterial lactate [mmol/l]		4.9 [2.5–8.6]	4 [2.1–7.1]	5.3 [3.1–10.1]	**0.007**
Confusion at presentation		61 (37%)	33 (34%)	28 (41%)	0.28
Previous myocardial infarction or CABG		39 (23%)	23 (24%)	16 (23%)	0.94
LV-EF prior to Impella		20 [15-30]	20 [15-30]	20 [15-25]	0.21
Systolic blood pressure at presentation		80 [70–102]	80 [70–110]	80 [65–100]	0.59
**Infarct localization**					
	LMCA	43 (26%)	26 (27%)	17 (25%)	0.86
	LAD	78 (47%)	46 (47%)	32 (46%)	0.22
	LCX	17 (10%)	11 (11%)	6 (9%)	0.32
	RCA	25 (15%)	14 (14%)	11 (16%)	0.61
	Bypass graft	3 (2)	0 (0%)	3 (4%)	
Liver failure		20 (12%)	13 (13%)	7 (10%)	0.37
Intubated before procedure		119 (72%)	61 (63%)	54 (78%)	**0.03**

*Data are presented as n (%), mean ± SD, or median plus interquartile ranges. CABG, coronary artery bypass grafting; eGFR, estimated glomerular filtration rate; LAD, left anterior descending artery; LCX, left circumflex coronary artery; LMCA, left main coronary artery; LV-EF, left-ventricular ejection fraction; PCI, percutaneous coronary intervention; RCA, right coronary artery. p-values are displayed in bold if statistically significant*.

### Impella Initiation Pre- vs. Post-PCI

**Figure 3:**
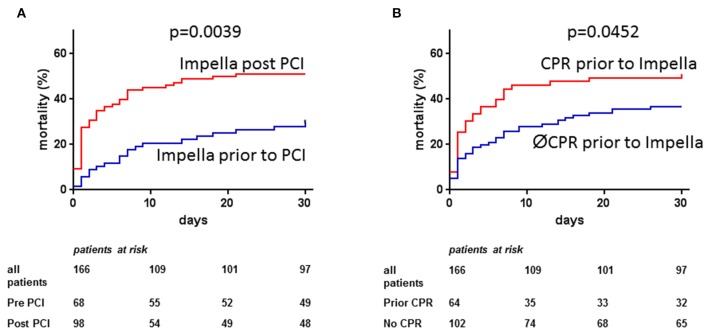
Observed 30-day mortality in acute myocardial infarction cardiogenic shock (AMI-CS) on Impella: Observed 30-days mortality in AMI-CS treated with Impella was lower if implantation was performed prior to percutaneous coronary intervention (PCI, **A**) and higher in patients with cardiac arrest prior to Impella implantation **(B)**.

Overall 30-day mortality was higher when Impella was implanted post-PCI (51%, *n* = 50/98) compared to when Impella was implanted pre-PCI (28%, *n* = 19/68; *p* = 0.0039, Figure 3A). Mortality risk score values and their distribution were similar in both groups (median CardShock score of 5 in both groups; median IABP-Shock II score of 3 in both groups; Supplemental Figures 1A,B). Patients receiving Impella following PCI had higher admission lactate levels (6.9 ± 5.2 vs. 5.1 ± 3.6 mmol/l, *p* = 0.0178). However, both groups were of similar age (65 ± 11 vs. 67 ± 13 years, *p* = 0.2618), had comparable renal function (eGFR 39 ± 24 vs. 43 ± 24 ml/min, *p* = 0.3410) and comparable LV function prior to support (21 ± 11% vs. 21 ± 11%, *p* = 0.9534) (Supplemental Table 1).

With regard to the interaction between risk scores and timing of PCI, survival was better than predicted in all categories when Impella was placed prior to PCI (Figures 1B, 2B). Furthermore, with the exception of IABP-II Shock high-risk group, which included a very small number of patients (*n* = 15), survival trended to be better when Impella support was initiated pre-PCI instead of post-PCI.

### Impact of Cardiac Arrest on Survival

Thirty-day mortality was higher in resuscitated patients (50%, *n* = 32/64) vs. non-resuscitated patients (36%, *n* = 37/102; *p* = 0.0452, Figure 3B). Mortality risk score values were numerically higher in the resuscitated vs. non-resuscitated patients (CardShock score 6 vs. 5; IABP-Shock II score 3 vs. 2). Resuscitated patients were younger (62 ± 12 vs. 68 ± 11 years, *p* = 0.001) and had higher admission lactate levels (8.6 ± 5.2 vs. 4.6 ± 3.7 mmol/l, *p* < 0.0001), but similar renal function (eGFR 41 ± 23 vs. 39 ± 23 ml/min, *p* = 0.6491). The distribution of risk scores was similar for patients with or without prior resuscitation (Supplemental Figures 1C,D).

In all risk groups, the presence of cardiac arrest was associated with worse outcome, particularly in patients with high IABP-II Shock risk scores. In the absence of cardiac arrest, 30-day mortality rate was lower than predicted by the respective score in cases with highest predicted risk, similar in those with predicted intermediate risk, and numerically higher in those with low predicted risk (Figures 1C, 2C). Very high admission lactate levels (>10 mmol/L) appear to be associated with high mortality particularly in patients without prior cardiac arrest (Supplemental Figure 2).

### Univariate and Multivariate Analyses

Results of univariate analysis (Table 4) showed that both 12- and 30-day mortality rates were associated with the CardShock score, the CardShock category, IABP-Shock II score (but not the IABP-Shock II risk category), whether patients had been resuscitated and whether Impella was placed prior to PCI. In multi-regression analysis, only risk scores and the timing of Impella placement remained statistically significant (Table 4). Interestingly, adding IABP-Shock II score to the CardShock score did not enhance the ability to discriminate between survivors and non-survivors (Supplemental Figure 3, Supplemental Table 2).

**Table 4:** Multivariate logistic regression analysis predicting mortality.

**Parameter**	**12 day mortality**		**30 day mortality**	
	**Univariate regression**		**Univariate [HR (95% CI)]**	
	**HR (95% CI)**	***P***	**HR (95% CI)**	***P***
Card Shock score	1.61 (1.22–2.13)	**0.001**	1.58 (1.21–2.05)	**0.001**
Card Shock category	2.02 (1.21–3.38)	**0.007**	1.94 (1.19–3.15)	**0.008**
IABP Shock score	1.29 (1.02–1.62)	**0.03**	1.27 (1.02–1.59)	**0.03**
Resuscitation before	2.06 (1.07–3.94)	**0.03**		0.057
Impella implantation before PCI	0.33 (0.16–0.67)	**0.002**	0.42 (0.22–0.80)	**0.009**
	**Multivariate regression**		**Multivariate regression**	
Card Shock score	1.64 (1.22–2.19)	**0.001**	1.57 (1.20–2.06)	**0.001**
Resuscitation before	1.54 (0.76–3.11)	0.23	1.44 (0.73–2.84)	0.290
Impella implantation before PCI	0.31 (0.15–0.64)	**0.002**	0.42 (0.21–0.82)	**0.012**
Card Shock category	1.97 (1.15–3.37)	**0.013**	1.88 (1.14–3.10)	**0.013**
Resuscitation before	1.69 (0.85–3.83)	0.13	1.58 (0.81–3.1)	0.18
Impella implantation before PCI	0.33 (0.16–0.68)	**0.003**	0.44 (0.23–0.86)	**0.016**
IABP Shock score	1.27 (1.001–1.61)	**0.049**	1.26 (1.002–1.57)	**0.048**
Resuscitation before	1.76 (0.88–3.49)	0.11	1.63 (0.84–3.15)	0.15
Impella implantation before PCI	0.33 (0.16–0.67)	**0.02**	0.43 (0.22–0.83)	**0.013**

*p-values are displayed in bold if statistically significant*.

## Discussion

Results from adequately powered prospective trials are still lacking to guide the use of Impella or any other MCS devices beside IABP in CS patients and current practice is predominantly based on individual experience (26). To provide additional insights, we combined data from four high-volume European cardiogenic shock centers highly experienced in the use of Impella to treat AMI-CS. First, in patients meeting criteria for the IABP-Shock II study, we observed that Impella use was associated with lower mortality than predicted in patients deemed at high risk based predominantly on assessment using a validated risk score, the CardShock score. Patients considered high risk accounted for 49% of the overall cohort. Second, 30-day mortality trended to be lower when Impella was implanted prior to, rather than after PCI, predominantly in patients at lower predicted risk potentially indicating a more modifiable or reversible stage of shock or prevention of reperfusion injury driven compromise. Third, patients who experienced cardiac arrest trended to have higher mortality compared with those who never arrested, which was observed in all risk categories. Finally, in multi-regression analysis factors that were independently associated with higher mortality were higher CardShock- or IABP Shock II-scores, pre-implantation arrest and Impella implantation after PCI. Older age and increased lactate differed between survivors and non-survivors, but their association with mortality was not included in the multi-regression model since they are both incorporated into the CardShock and IABP-Shock II scores.

Pre-Impella cardiac arrest is a known contributor to AMI-CS mortality (17, 27). Thirty-nine percentage of our patients experienced cardiac arrest prior to Impella which was higher than the 28% of patients in the original CardShock population but less than the ~45% of patients in IABP-Shock II. However, neither CardShock nor IABP-Shock II scores include prior arrest as risk factors. Lactate levels tend to be higher in patients following cardiac arrest and this is included in the risk scores. However, cardiac arrest is included in the recently introduced SCAI classification system for CS (28).

Several factors contribute to mortality in post-arrest CS patients. In addition to deranged total body metabolism and organ dysfunction, a major contributor to mortality is anoxic brain damage that has occurred prior to hospital admission and prior to insertion of a hemodynamic support device (17). This impacted the IMPRESS-in-SEVERE-SHOCK trial, which failed to demonstrate a benefit from Impella support in a population with AMI-CS in which 92% of patients were post-arrest (12). While that trial was interpreted as showing non-effectiveness of Impella support in CS, it was a study that recruited a low number of patients with potentially salvageable neurologic function. The IABP-Shock II entry criteria (applied in the present study) excluded patients who had undergone resuscitation for more than 30 min or were in a coma with fixed dilatation of pupils. Whether any form of circulatory support might change the overall outcome in patients following cardiac arrest was beyond the scope of our analysis. The higher rate of resuscitated patients in our analysis might have impacted the findings on observed compared to predicted risk in particular when using the CardShock score. In clinical practice, however, it will be difficult to withhold any circulatory support device from an AMI-CS patient only due to prior resuscitation when no sufficient tool exists to predict the potential neurological damage caused by cardiac arrest. On an individual basis, it might be feasible to use circulatory support devices in those patients after cardiac arrest, in whom extensive neurological damage is unlikely.

In addition to cardiac arrest, timing of Impella implantation (pre- vs. post-PCI) has been noted in several retrospective analyses to be related to better outcomes (13–16). In our cohort, Impella implantation pre-PCI seemed to be associated with better outcomes in the low/intermediate-risk groups (Figures 1B, 2B). A hypothesis raised by this observation may be that Impella pre-PCI vs. post-PCI may help with reperfusion injury and infarct expansion in early phases of AMI-CS. Of note, there was no significant difference in baseline characteristics between patients treated pre- vs. post-PCI, which is important since the decision of timing of implantation was not randomized. The consistency of our and prior independently obtained results indicating better outcomes with pre- vs. post-PCI Impella compared to those predicted by the CardShock score across risk categories, while very encouraging, should not be interpreted as proof that Impella use pre-PCI improves survival; this will require prospective studies. As ours was a retrospective analysis, we should be very careful considering a potential selection bias as confounder and have to explore the timing of Impella implantation in a controlled trial.

In the absence of prospective study results, there are several approaches to using existing registry databases to assess the impact of interventions, each with its own limitations. Recently, the authors contributed the current data to a larger analysis based on an attempted matching of baseline characteristics using patient-level data from the IABP-Shock II-trial (21). In contrast to all of the studies noted above, that study failed to detect an overall survival benefit of Impella, and had no advantage when Impella was used pre-PCI and no advantage when Impella was used in the absence of cardiac arrest. However, only 237 of the total 372 IABP-Shock II qualifying Impella patients could be matched. Despite matching, differences in baseline characteristics remained between groups. Given the small sample size, exclusion of a significant proportion (~40%) of Impella patients, existence of differences in baseline characteristics, uncertainty of comparability of risk between groups, uncertainty about composition of the population with regard to risk all limit the generalizability of those findings. Each of these factors is potentially important but, in particular, the current analysis points out the importance of understanding the selected population's overall risk of mortality. Another potential advantage of the present study is that all participating University hospitals use a similar dedicated algorithm for recognizing, diagnosing, and treating CS patients.

Knowing that randomly selecting patients for such an intended matched-pair analysis might not provide an ideal control group, we chose an alternate approach of calculating score-based predicted mortality risk on an individual patient level and compared the average observed event rate with predicted risk. Thereby, all qualifying patients form the registry were included and, by using the validated scores, in essence compared to information from all patients from the IABP-Shock II and CardShock studies. In our analysis we found that a potential benefit of Impella might be observed in very sick CS patients categorized as high-risk by CardShock score. Whether selecting patients for Impella treatment based on CardShock score assessment will help to reduce mortality in AMI-CS, however, has to be tested prospectively. Trying to do a propensity matched statistical study design to create a control population from the IABP-Shock II database, ideally with a greater ratio of control: Impella patients to avoid bias, would have likely resulted in even less patients included in the analysis.

More recently, others have used data from larger American databases for cardiac catheterization and acute chest pain treatment (29). Of 28,304 patients with AMI-CS, they identified 1,768 treated with Impella and 8,471 treated with IABP within a 27 months period. In summary, Impella patients in that analysis were significantly sicker and, therefore, a propensity matching was performed yielding 1,680 matched pairs. Despite matching, the primary analysis showed higher mortality and higher bleeding rates in the Impella compared to the IABP group. Hemodynamic parameters allowing for substantial assessment of shock and haemodynamic stabilization were not available, and no uniform definition of shock or indication for device implantation was used in this national registry, neither were both devices similarly available in the participating centers. Therefore, despite the huge number of patients the analysis is of limited value in terms of generalizability. Consequently, large randomized controlled trials are necessary to clarify the subject of Impella use in AMI-CS, an approach facilitated by the authors of this manuscript in the DanGer-Shock trial (20).

## Limitations

First, the present study utilized data from clinical practice registries, so neither control group nor randomization of treatment were available. Therefore, all results are, at most, hypothesis generating and cannot prove superiority of Impella in AMI-CS. As noted above, an alternate approach could have employed propensity score-matching to select patients from an existing database of patients with known baseline clinical characteristics and risk factors for mortality (e.g., the IABP-Shock study database) to create a control group with characteristics and risk factors similar to those of the current cohort which was subjected to a different treatment strategy. However, assessing heterogeneous patient cohorts such as those encountered in CS would require a much larger database for propensity matching based on all essential parameters such as age, sex, left-ventricular ejection fraction, renal function, admission lactate, prior resuscitation, timing of initiation of support relative to PCI and mechanical ventilation. As also noted, a properly powered prospective randomized controlled trial comparing Impella vs. standard of care in AMI-CS, the DanGer study (20) is highly anticipated. Until such a trial is finished, retrospective analyses like ours might suggest to use Impella in properly selected patients; even so the statistical differences were small, our data—derived from tertiary centers with strict shock protocols- suggest to use Impella rather in higher than lower risk patients.

## Conclusions

In the absence of randomized trails, the decision whether and which form of MCS to be used is based on institutional and clinical experience. While our data do not support unselected use of circulatory support in AMI-CS, identifying patients deemed at higher risk using scores such as the IABP-Shock II and/or CardShock may help to select patients more likely to benefit from Impella treatment. In our cohort, CardShock score was more predictive of outcomes.

## Data Availability Statement

The datasets generated for this study are available on request to the corresponding author.

## Ethics Statement

The studies involving human participants were reviewed and approved by Hannover Medical School. Written informed consent for participation was not required for this study in accordance with the national legislation and the institutional requirements.

## Author Contributions

AS, NW, RW, and JM designed the study. All authors acquired and analyzed the data. AS, NW, DB, RW, and JM drafted the manuscript. J-TS, AZ, MM, and NJ critically revised the manuscript. All authors agree to be accountable for all aspects of the work ensuring that questions related to the accuracy or integrity of any part of the work are appropriately investigated and resolved.

## Conflict of Interest

AS, NW, DB, RW, and JM have received modest lecture fees, honoraria and research grants from Abiomed. The remaining authors declare that the research was conducted in the absence of any commercial or financial relationships that could be construed as a potential conflict of interest.

## Abbreviations

AMI: acute myocardial infarction
CS: cardiogenic shock
ECMO: extracorporeal membrane oxygenation
HACURE: HAnnover Cardiac Unloading Registry
IABP: intraaortic balloon pump
LV: left ventricle/left ventricular
MCS: mechanical circulatory support
NSTEMI: non-ST-segment elevation myocardial infarction
PCI: percutaneous coronary intervention
STEMI: ST-segment elevation myocardial infarction.
