# Supplementary material for: Influence of Timing and Predicted Risk on Mortality in Impella-Treated Infarct-Related Cardiogenic Shock Patients
Source: Front Cardiovasc Med. 2020 May 14;7:74. doi: 10.3389/fcvm.2020.00074 (PMC7240000; doi:10.3389/fcvm.2020.00074)
Supplement: Supplementary file 1 [file Data_Sheet_1.pdf]

Influence of Timing and Predicted Risk on Mortality in Impella-Treated Infarct-Related Cardiogenic Shock Patients

Supplement

Andreas Schäfer, MD[a*], Nikos Werner, MD[bc*], Daniel Burkhoff[d], MD, PhD, Jan-Thorben Sieweke, MD[a], Andreas Zietzer, MD[b], Maryna Masyuk, MD[e], Nanna Louise Junker Udesen[f], Ralf Westenfeld, MD[e*], Jacob Eifer Møller, MD[f*]

[a] Cardiac Arrest Center & Advanced Heart Failure Unit, Department of Cardiology and Angiology, Hannover Medical School, Hannover, Germany
[b] Department of Cardiology, University Heart Center, Bonn, Germany
[c] Department of Cardiology, Heart Center Trier, Krankenhaus der Barmherzigen Brüder, Trier, Germany
[d] Cardiovascular Research Foundation, New York, NY, USA
[e] Department of Cardiology, Pulmonology and Vascular Medicine, Heinrich Heine University, Düsseldorf, Germany
[f] University Hospital Odense, Department of Cardiology, Odense, Denmark
*These authors contributed equally to the manuscript

Running title: Impella in AMI-Cardiogenic Shock

Manuscript words:	4525
Abstract words:	236
References:	29
Figures:	3
Tables:	4

Supplementary Figure 1: Frequency distribution of score values depending on the type of Impella: Relative frequency of individual score values for IABP Shock II-score (A&C) and CardShock-score (B&D) stratified by implantation before/after revascularization (A&B) and resuscitation prior to Impella implantation (C&D).

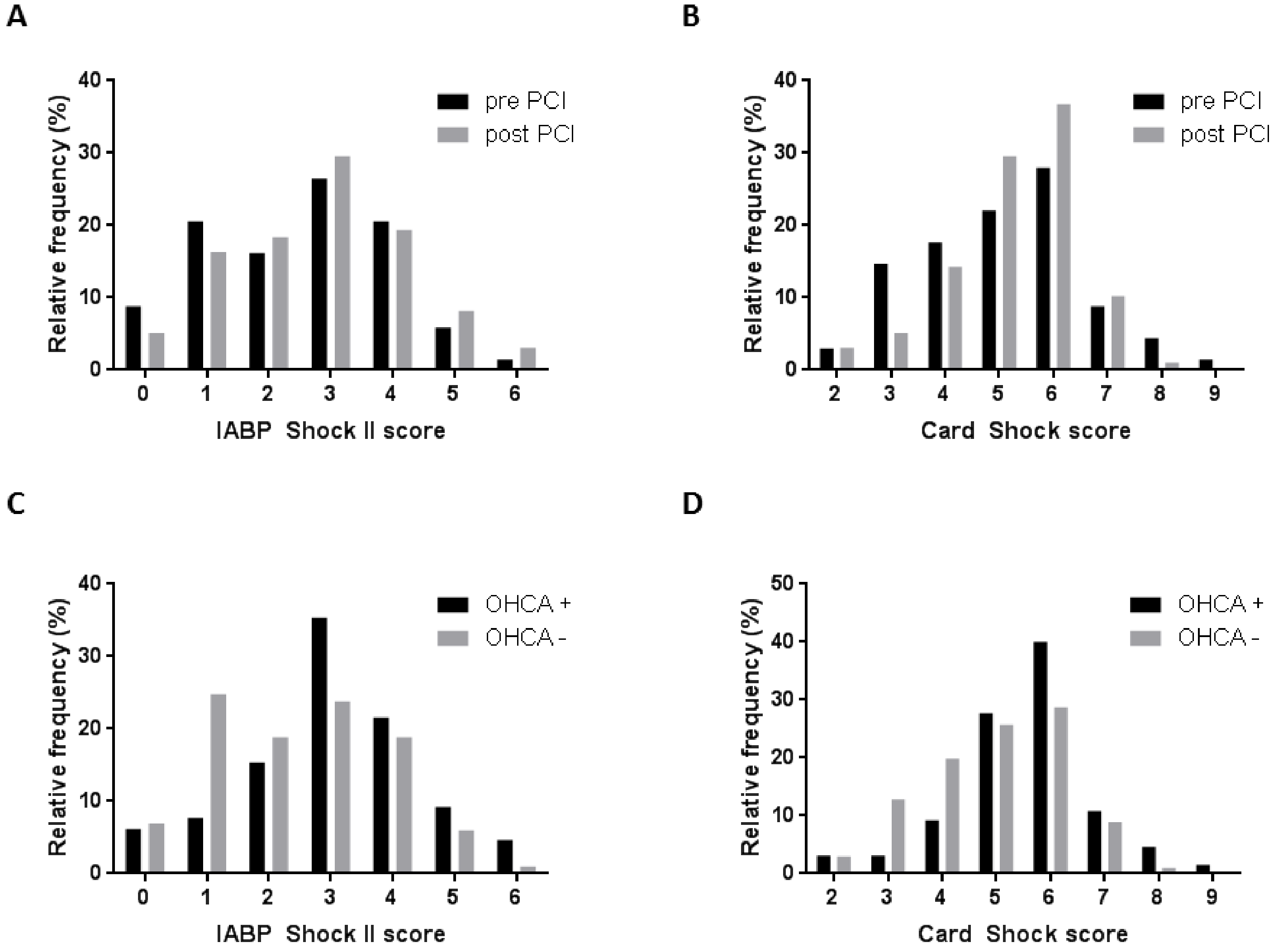

Suppl. Fig. 1

Supplementary Figure 2: Influence of admission lactate on 30-day mortality in acute myocardial infarction cardiogenic shock (AMI-CS) on Impella: Influence of admission lactate levels on mortality are displayed separated for presence or absence of cardiac arrest prior to Impella implantation.

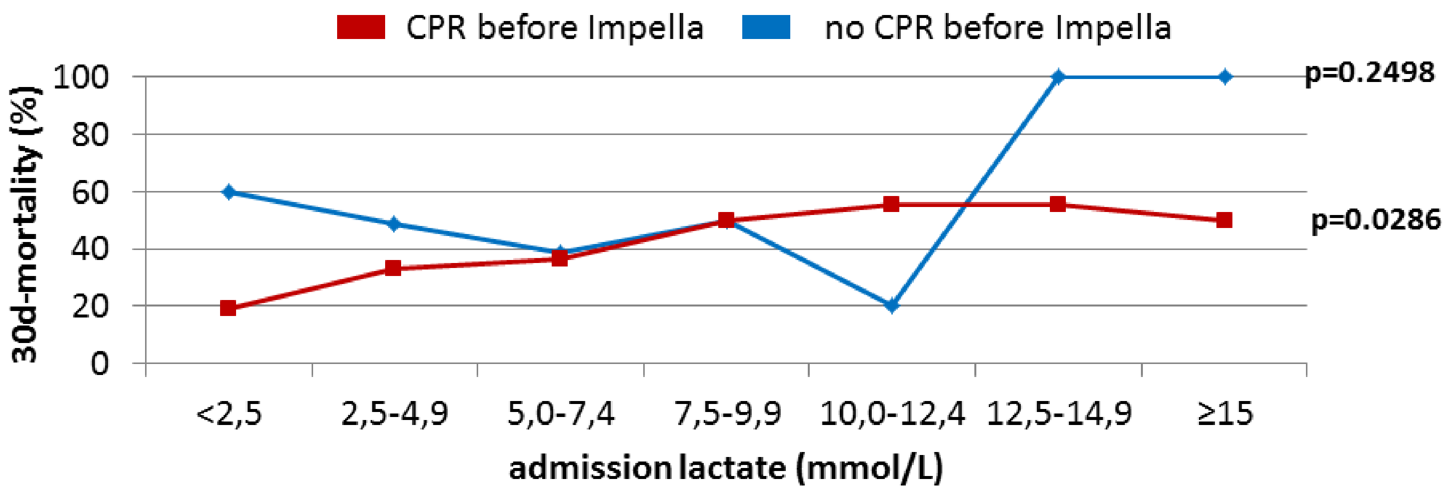

Suppl. Fig. 2

Supplementary Figure 3: receiver operating characteristic (ROC) curve for Card Shock Score, IABP Shock Score and the combination of both. The area under the curve of Card Shock Score and the combination of IABP Shock Score and Card Shock Score are identical.

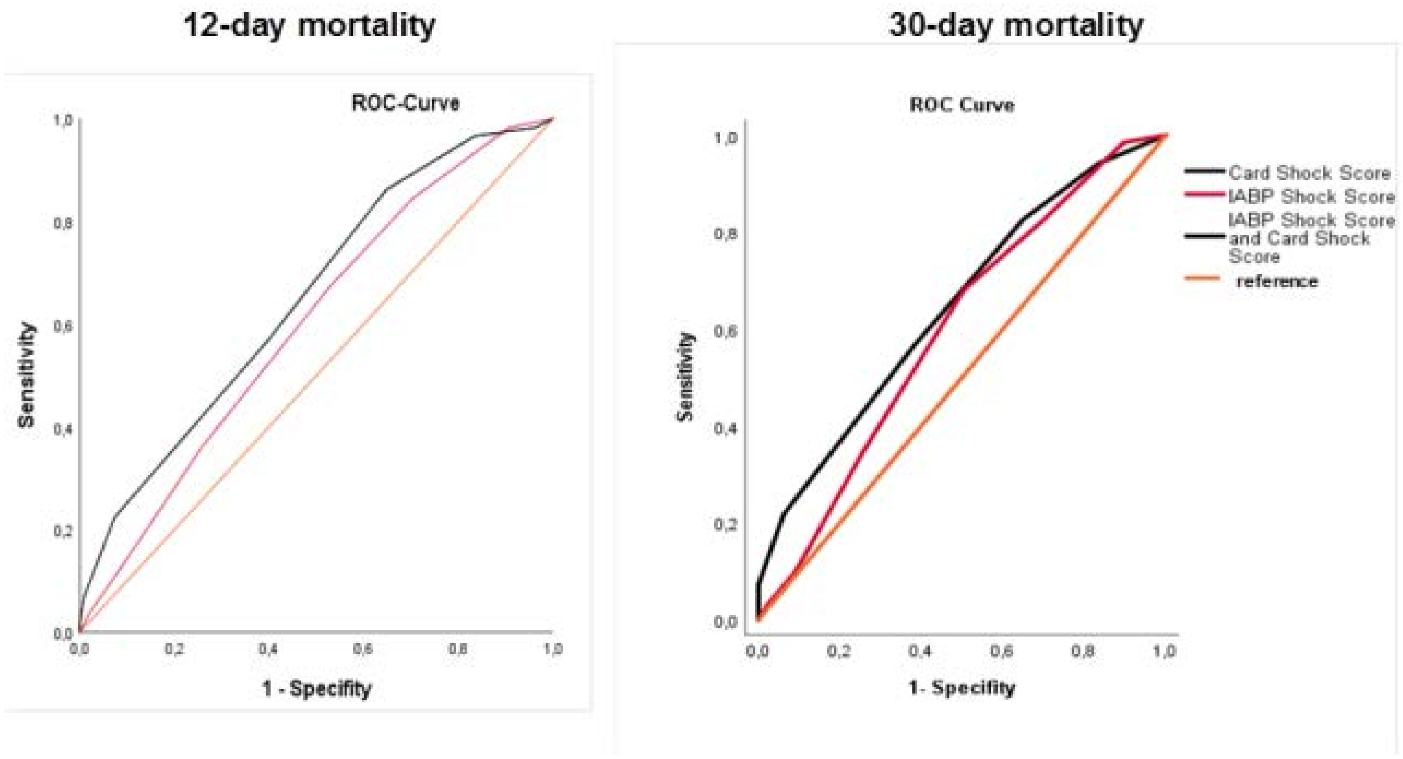

Suppl. Fig. 3

Suppl. Table 1: Characteristics and Outcome from the IABP-Shock II-like patients depending on time of Impella implantation before vs after PCI

	Impella pre-PCI<br>n= 98<br>mean±SD, median [IQR], n (%)	Impella post PCI<br>n=68<br>mean±SD, median [IQR], n (%)	p-value<br>pre vs. post
Age, mean (SD), years	67 ± 13	65 ± 11	0.2618
Cardiac arrest prior to Impella	36 (37%)	28 (41%)	0.4903
Use of Impella CP	73 (74%)	54 (79%)	0.3623
Admission lactate, mean (SD), mmol/L	5.1 ± 3.6	6.9 ± 5.2	0.0178
eGFR, mean (SD), ml/min	43 ± 24	39 ± 24	0.3410
LV-EF prior to Impella, mean (SD), %	21 ± 11	21 ± 11	0.9534
IABP Shock II Score, median (IQR)	3 [1-4]	3 [2-4]	0.2778
CardShock Score, median (IQR)	5 [4-6]	5 [5-6]	0.4085
Duration of shock prior to Impella, mean (SD), min	485±1320	328±523	0.3371

eGFR-estimated glomerular filtration rate; LV-EF-left-ventricular ejection fraction; SAPS II-Simplified Acute Physiology Score-II

Suppl. Table 2: To test the discriminative ability of the prediction model for mortality we performed an area under the receiver operating characteristic (ROC) curve for Card Shock Score, IABP Shock Score and the combination of IABP Shock Score and Card Shock Score as provided in Suppl. Figure 3

12-day mortality

Variable	AUC	95% CI
Card Shock Score	0.650	0.564-0.735
IABP Shock Score	0.598	0.510-0.686
IABP Shock Score and Card Shock Score	0.650	0.564-0.735

30-day mortality

Variable	AUC	95% CI
Card Shock Score	0.645	0.560-0.729
IABP Shock Score	0.597	0.511-0.680
IABP Shock Score and Card Shock Score	0.645	0.560-0.729
